# Silicone Rubber Fabry-Perot Pressure Sensor Based on a Spherical Optical Fiber End Face

**DOI:** 10.3390/s22051862

**Published:** 2022-02-26

**Authors:** Changxing Jiang, Xiaohua Lei, Yuru Chen, Shaojie Lv, Xianming Liu, Peng Zhang

**Affiliations:** Key Laboratory of Optoelectronic Technology & Systems, Education Ministry of China, Chongqing University, Chongqing 400044, China; 201908021007@cqu.edu.cn (C.J.); chenyuru@cqu.edu.cn (Y.C.); 20172469@cqu.edu.cn (S.L.); xmliu@cqu.edu.cn (X.L.); zhangpeng@cqu.edu.cn (P.Z.)

**Keywords:** pressure, Fabry-Perot sensor, silicone rubber, spherical optical fiber

## Abstract

To improve the fringe contrast and the sensitivity of Fabry-Perot (FP) pressure sensors, a silicone rubber FP pressure sensor based on a spherical optical fiber end face is proposed. The ratio of silicone rubber ingredients and the diameter and thickness of silicone rubber diaphragm were optimized by a simulation based on experimental tests that analyzed elastic parameters, and the influence of the radius of a spherical optical fiber and the initial cavity length of the sensor on the fringe contrast was investigated and optimized. Pressure sensor samples were fabricated for pressure test and temperature cross-influence test. Gas pressure experimental results within a pressure range of 0~40 kPa show the average sensitivity of the sensor is −154.56 nm/kPa and repeatability error is less than 0.71%. Long-term pressure experimental results show it has good repeatability and stability. Temperature experimental results show its temperature cross-sensitivity is 0.143 kPa/°C. The good performance of the proposed FP pressure sensor will expand its applications in biochemical applications, especially in human body pressure monitoring.

## 1. Introduction

The optical fiber FP pressure sensor has many advantages, such as anti-electromagnetic interference, small size, etc. It has been gradually used in aerospace, bridge detection, large instrument, equipment monitoring, etc. [1,2,3,4]. Recent studies show it has also potential biochemical applications, especially in human body pressure monitoring [5,6,7,8,9]. For example, Shen Liu et al. proposed an arc discharge technology to produce an ultrathin silica diaphragm with a thickness of only 173 nm, and its pressure sensitivity is 12.22 nm/kPa [10]. Xu Guo et al. proposed a thermal bonding technology, in which only a 1.2 μm thickness silicon dioxide diaphragm bonded to the end face of single-mode fiber, where there is an etched microcavity. Additionally, its static pressure sensitivity is 12.4 nm/kPa [11]. In addition, a few layers of graphene material has also been used to improve the pressure sensitivity to 39.4 nm/kPa [12]. It can be seen that the effective way to achieve high-pressure sensitivity is to reduce the thickness of the diaphragm. However, the excessively thin diaphragm is too fragile, which makes the manufacturing process difficult, significantly increases manufacturing costs, and reduces its reliability [13,14,15,16,17].

To solve this problem, some FP sensors with diaphragms based on new elastic materials have been reported. To date, Jushuai Wu et al. has reported the smallest FP pressure sensor, with a SU-8 photoresist directly coated on the end face of a single-mode optical fiber. It has a support structure diameter of 100 μm and a diaphragm thickness of 11 μm and shows a sensitivity of 2.93 nm/MPa in the range of 0~700 kPa [18]. Cheng Luo et al. used a PDMS polymer as the pressure sensing diaphragm; its thickness is 18.4 μm and pressure sensitivity is 100 pm/kPa [19]. Xin Cheng et al. investigated an FP pressure sensor based on a polymer of the silicone rubber diaphragm, with a thickness of 257 μm, and its pressure sensitivity is 0.69 nm/kPa [20]. From these studies, we can infer that the thickness of the polymer diaphragm can be 1–2 orders higher than that of the traditional diaphragm under the same sensitivity. Obviously, a thicker polymer diaphragm makes the manufacturing process easier. However, the FP sensors with polymer diaphragms also have low sensitivity and a low precision caused by the poor fringe contrast [21,22,23].

In this paper, we proposed a silicon rubber fiber FP pressure sensor based on a spherical end fiber. An FP cavity was formed by a spherical optical fiber end face and the silicone rubber surface. A convergence effect of the spherical surface increased the intensity of the reflected light from the rubber surface returning to the optical fiber, so the fringe contrast of the sensor greatly improved. Additionally, the sensitivity of the sensor also improved by optimizing the ratio of silicone rubber ingredients.

## 2. Fringe Contrast Analysis of FP Pressure Sensors

**Figure 1:**
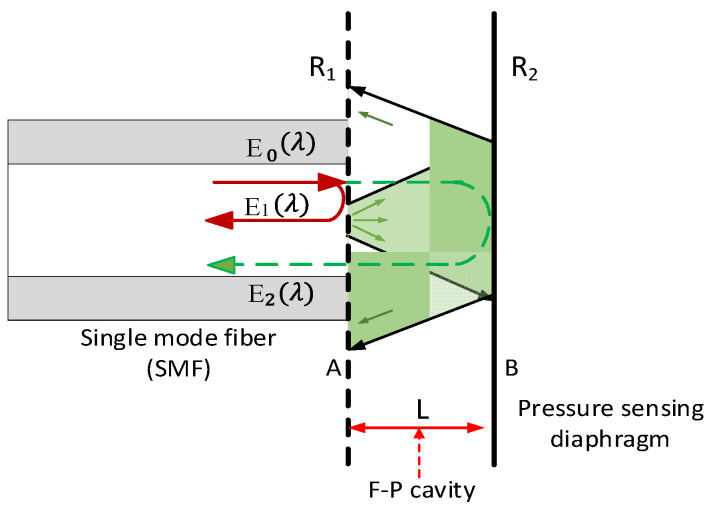
The optical path diagram of the optical fiber FP pressure sensor.

For a typical optical fiber FP pressure sensor, its optical path is shown in Figure 1. The incident light is ${\vec{E}}_{0}\left(\lambda \right)$; a part of the light reflected by the first surface *A* is recorded as ${\vec{E}}_{1}\left(\lambda \right)$, and the transmitted light reflected by the second surface *B* and then transmitted through the first surface is ${\vec{E}}_{2}\left(\lambda \right)$. The light reflected by surface *B* (pressure sensing diaphragm) cannot be completely coupled back to the optical fiber core because of the limited numerical aperture of optical fiber. As the cavity length *L* increases, the light that can be coupled back to the core decreases. When the diameter of the diaphragm is equivalent to the diameter of the optical fiber, the pressure applied on the diaphragm will increase its curvature, which will increase the loss of light coupled back into the fiber. This increases the energy difference between the ${\vec{E}}_{2}\left(\lambda \right)$ and the ${\vec{E}}_{1}\left(\lambda \right)$, reducing the fringe contrast of interference patterns and decreasing demodulation accuracy [24,25].

Suppose the transmission coefficient is η, which describes the total reflection loss from surface B to the optical fiber core. Effective reflectivity is defined as $\eta R_{2}=\eta r_{2}^{2}$, and expressions of ${\vec{E}}_{1}\left(\lambda \right)$ and ${\vec{E}}_{2}\left(\lambda \right)$ can then be written as Equations (1) and (2).

(1) $${\vec{E}}_{1}\left(\lambda \right)=\sqrt{R_{1}}{\vec{E}}_{0}\left(\lambda \right)$$

(2) $${\vec{E}}_{2}\left(\lambda \right)={\left(1-\sqrt{R_{1}}\right)}^{2}\eta \sqrt{R_{2}}{\vec{E}}_{0}\left(\lambda \right)\cdot \exp\left(j\frac{4\pi L}{\lambda }\right)$$

The total electric field ${\vec{E}}_{R}\left(\lambda \right)$ can be expressed as

(3) $${\vec{E}}_{R}\left(\lambda \right)={\vec{E}}_{1}\left(\lambda \right)+{\vec{E}}_{2}\left(\lambda \right)=\left[\sqrt{R_{1}}+{\left(1-\sqrt{R_{1}}\right)}^{2}\eta \sqrt{R_{2}}\cdot \exp\left(j\frac{4\pi L}{\lambda }\right)\right]\cdot {\vec{E}}_{0}\left(\lambda \right)$$

Then, the intensity $I_{R}\left(\lambda \right)$ can be written as

(4) $$I_{R}\left(\lambda \right)={\vec{E}}_{R}\left(\lambda \right)\cdot {\vec{E}}_{R}^{*}\left(\lambda \right)=\left[\frac{R_{1}+\eta R_{2}+2\eta \sqrt{R_{1}R_{2}}cos\frac{4\pi L}{\lambda }}{1+\eta R_{1}R_{2}+2\eta \sqrt{R_{1}R_{2}}cos\frac{4\pi L}{\lambda }}\right]\cdot I_{0}\left(\lambda \right)$$

Let the fringe contrast of interference pattern be

(5) $$K=\frac{I_{R_{max}}-I_{R_{min}}}{I_{R_{max}}+I_{R_{min}}}$$

**Figure 2:**
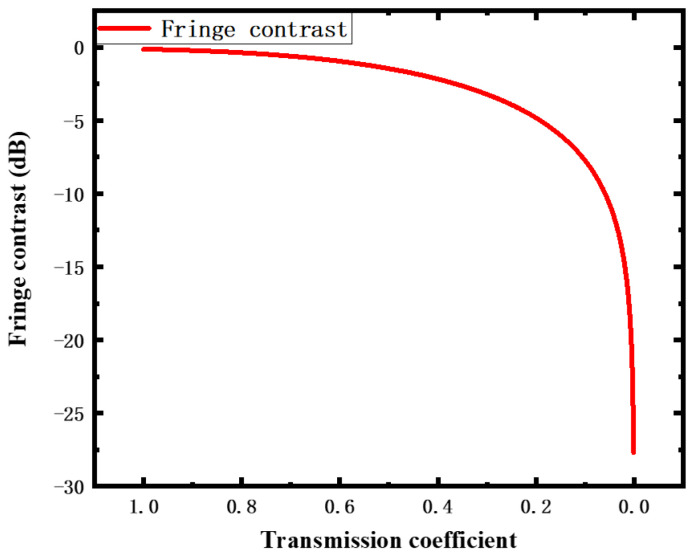
Relationship between transmission coefficient and fringe contrast of interference pattern.

To further understand the relationship between the transmission coefficient and the fringe contrast, a simulation was carried out based on Equations (4) and (5), which is shown in Figure 2. The fringe contrast decreases with the decrease in transmission coefficient because of the high reflection loss caused by the change in the cavity length *L* or the diaphragm curvature. The reduction in fringe contrast leads to the deterioration of the signal-to-noise ratio and low demodulation accuracy. Therefore, it is necessary to investigate a new sensor structure to increase the intensity of ${\vec{E}}_{2}\left(\lambda \right)$ so that the fringe contrast can be improved.

## 3. Sensor Design Based on a Spherical End Face

### 3.1. Sensor Structure

**Figure 3:**
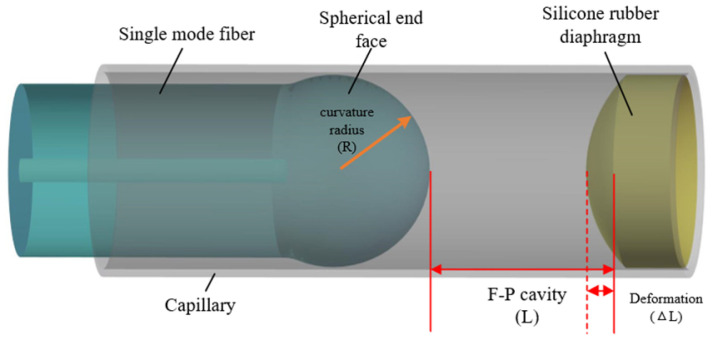
Structure diagram of the proposed sensor based on a spherical end face.

In order to improve the fringe contrast, a silicone rubber FP pressure sensor structure based on a spherical end face was proposed, and the sensor structure is shown in Figure 3.

The sensor was composed of single-mode optical fiber with a spherical end face, a quartz capillary, and a silicone rubber diaphragm. The FP cavity was formed by the spherical end face and the inner surface of the silicone rubber. When the light is reflected by the silicone rubber diaphragm, more light rays will be coupled back into the fiber by the convergence effect of the spherical surface [26,27,28]. It also can be seen from Figure 3 that the performance of the diaphragm is related to the sensitivity of the proposed sensor and is affected by the material of silicone rubber. Therefore, further analysis is needed to optimize the parameters of the silicone rubber diaphragm to ensure high sensitivity, and to optimize the parameters of a spherical optical fiber, and initial cavity length of the sensor to improve the fringe contrast.

### 3.2. Parameters Optimization of Silicone Rubber Diaphragm

As silicone rubber diaphragms with different parameters have variable mechanical properties, static modeling is required to study the deformation of diaphragms with different parameters under different pressures. Silicone rubber consists of components A and B. The main component of A is the silicone resin, which has good insulation, temperature resistance, and water resistance; Component B is mainly composed of a crosslinking agent and curing catalyst. The silicone contained in component A will affect the crosslinking density and strength of silicone rubber. Properly increasing silicone resin can significantly improve the actual mechanical properties of silicone rubber formed after curing. The crosslinking agents contained in component B can effectively improve the tear strength of silicone rubber. The mass ratio of components A and B will affect the mechanical properties of the cured material. Therefore, the mass ratio is an important factor affecting the performance of silicone rubber diaphragms [29].

To optimize the performance of silicone rubber diaphragm, the Mooney–Rivlin model, which is suitable for analyzing hyper elastomers with incompressibility and high deformation, was used to describe the mechanical properties of the silicone rubber [30].

Assume the deformation of the silicone rubber is less than 150%; here, the Mooney–Rivlin model can be simplified with two parameters, and the relationship between stress value and the tensile ratio is derived, according to [31], as follows:

(6) $$\frac{\sigma }{2\left(k-1/k^{2}\right)}=C_{10}+C_{01}/k$$

where *σ* refers to the stress value, and *k* is the tensile ratio. Additionally, the tensile ratio *k* is defined as

(7) $$k=\frac{L+\Delta L}{L}$$

where *L* refers to the initial length of the silicone rubber sample, and Δ*L* refers to the deformation. 

From Equation (6), it can be derived that $C_{10}$ and $C_{01}$ coefficients affect the deformation performance of silicone rubber. To determine these two coefficients, three samples, which have the same size of 25 mm × 6 mm × 3.6 mm, with a ratio of components A and B of 6:1, 8:1, and 10:1, respectively, were prepared and tested by the uniaxial tensile test.

**Figure 4:**
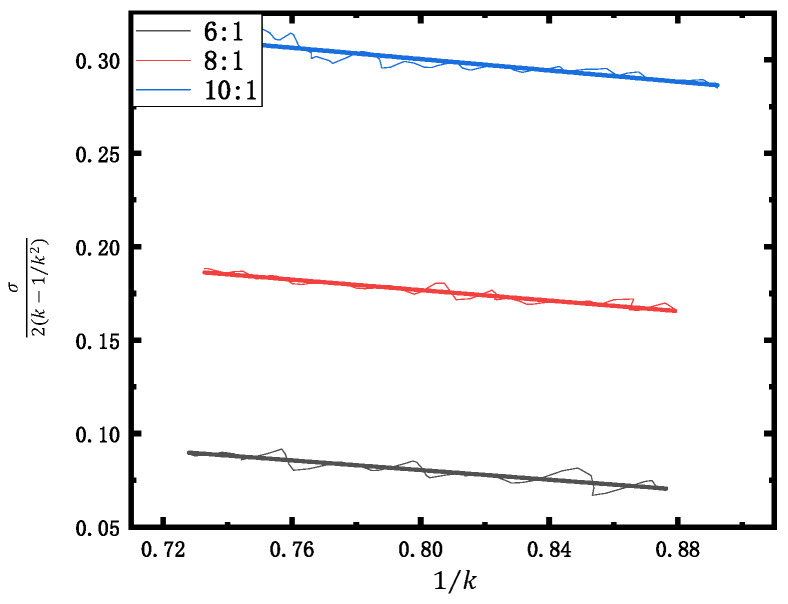
Relationship between tensile ratio and stress.

Relationship between tensile ratio (1/k) and stress σ/(2(k−1⁄k^2)) obtained from the tensile test was plotted, which is shown in Figure 4. According to Equation (6), the slope and intercept of the fitted line function were extracted to calculate $C_{10}$ and $C_{01}$ coeeficients. The results are shown in Table 1.

**Figure 5:**
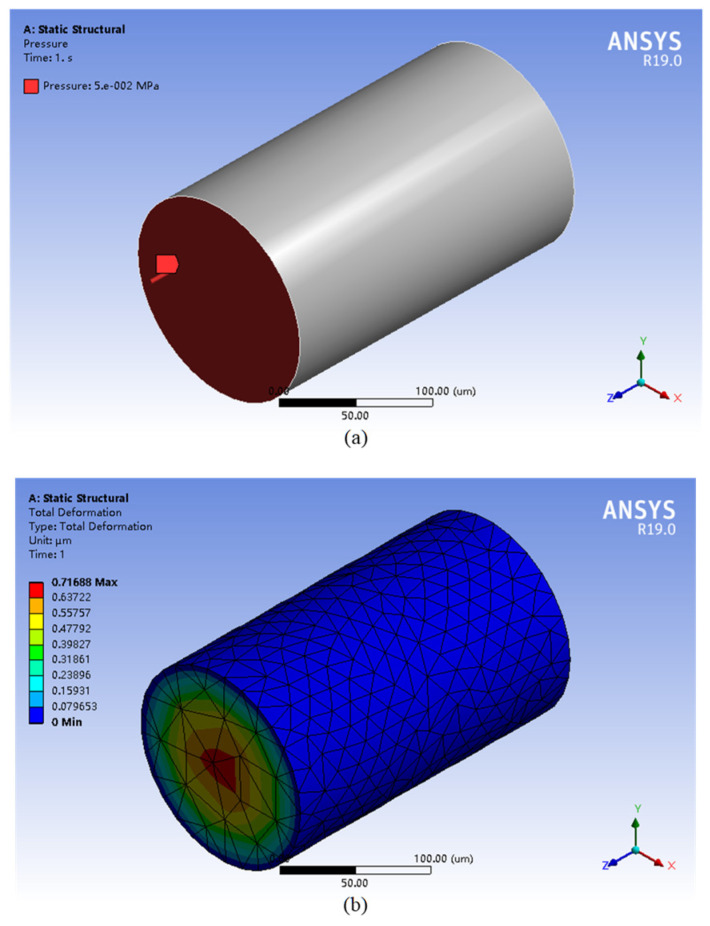
Model building diagram: (**a**) simulation model diagram and (**b**) deformation cloud diagram.

As the relationship between pressure and deformation of silicone rubber diaphragm cannot be expressed directly by formula, the measured parameters *C*_10_ and *C*_01_ were used in a model built in ANSYS for simulation, as shown in Figure 5a. To simulate the situation of silicone rubber in the capillary, we set silicone rubber diaphragms with different diameters and thicknesses, and the boundary between the silicone rubber diaphragm and the inner surface of the capillary was fixed. The pressure range was set at 0~50 kPa. Mesh generation of the model is shown in Figure 5b.

**Figure 6:**
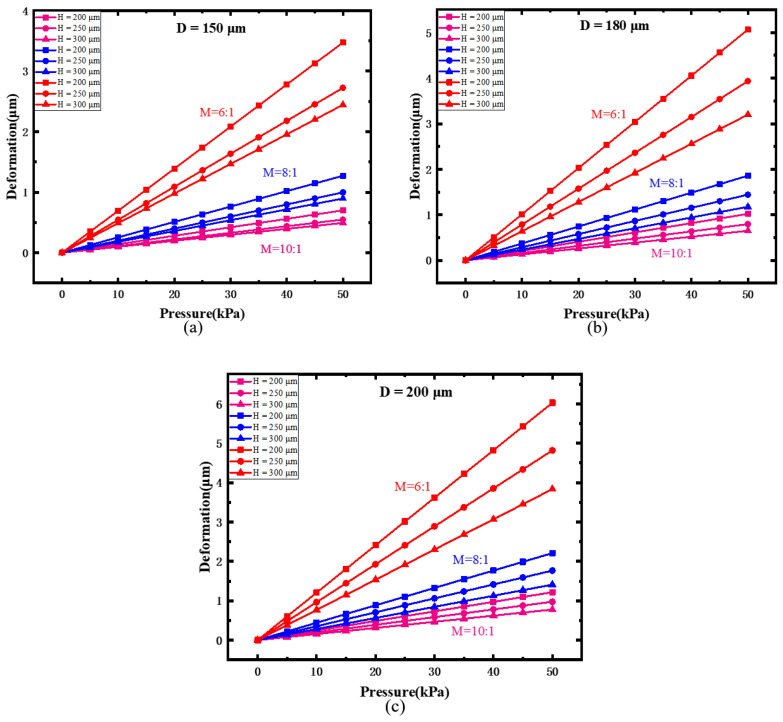
Relationship between the pressure and deformation: (**a**) diameter of 150 μm, (**b**) diameter of 180 μm, and (**c**) diameter of 200 μm.

The relationship between the pressure and deformation are plotted in Figure 6, with different thickness (H), diameter (D), and mass ratio (M). For example, when M = 6:1 and H = 200, with increasing diaphragm diameter, the deformation range slightly increases. When M = 6:1, and D = 150 μm, with increasing diaphragm thickness, deformation range increases. When H and D are fixed, the deformation range increases greatly. These results indicate that the mass ratio is a key parameter affecting the deformation performance of the silicone rubber diaphragm. Adjusting the mass ratio is an effective way to change the sensitivity of the silicone rubber diaphragm. It also can be seen that the deformation range of silicone rubber diaphragm under the pressure of 0~50 kPa is around several micros, which shows a better sensitivity, compared with reported diaphragms made by other elastic materials [18,19,20].

In fact, an excessive amount of the curing catalyst of component B will affect the reliability of the sensor and increase the complexity of the sensor fabrication process, so the parameters of mass ratio were selected to be 8:1. If the thickness of the diaphragm is too thin, the connection between the diaphragm and capillary will be weak, which may cause the slippage of the diaphragm in the capillary. If it is too thick, the sensitivity of the diaphragm will be reduced. Therefore, the thickness was selected to be 250 μm. A diameter of 150 μm of a capillary is too small for inserting a spherical end face fiber, a diameter of 200 μm is slightly large for sealing the gap between the capillary and the fiber. Therefore, the diameter of the capillary was selected to be 180 μm.

### 3.3. Optimization of the Spherical End Face

As the spherical end face is similar to the convex lens, its curvature radius and FP cavity length will affect the intensity of the final coupling efficiency, and therefore, the structural parameters of the sensor need to be optimized to achieve a better fringe contrast of interference pattern. 

**Figure 7:**
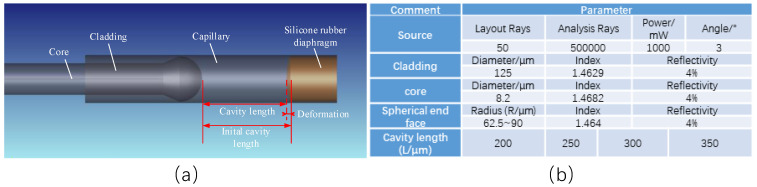
A 3D diagram of simulation model in ZEMAX. (**a**) the physical model of the silicone rubber pressure sensor; (**b**) simulation parameters.

The physical model of the silicone rubber pressure sensor established in ZEMAX is shown in Figure 7a, and parameters are given in Figure 7b. It can be seen that the FP cavity length change is equal to the initial cavity length minus the deformation of the silicone rubber diaphragm in the axial direction. Therefore, the pressure applied on the diaphragm was calculated by the deformation of the diaphragm in the axial direction, according to their relationship described by the results given in Figure 6.

**Figure 8:**
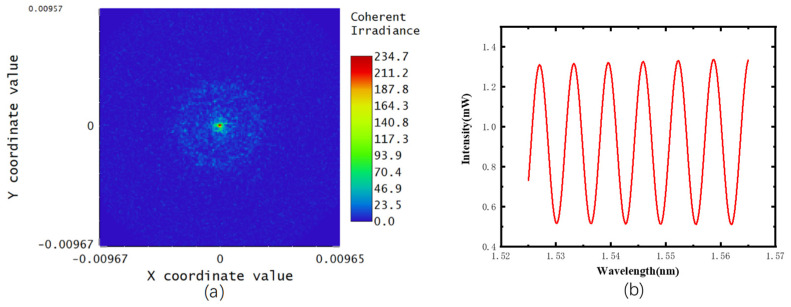
Ray tracing and wavelength scanning results. (**a**) simulation result for one single wavelength, (**b**) spectrum formed by simulation results of every wavelength within the range of 1525~1565 nm.

Ray tracing in ZEMAX could simulate the interference results for only one single wavelength at a time, shown in Figure 8a. The total interference intensity within the range of 1525~1565 nm was added by every simulation result of a single wavelength, shown in Figure 8b.

**Figure 9:**
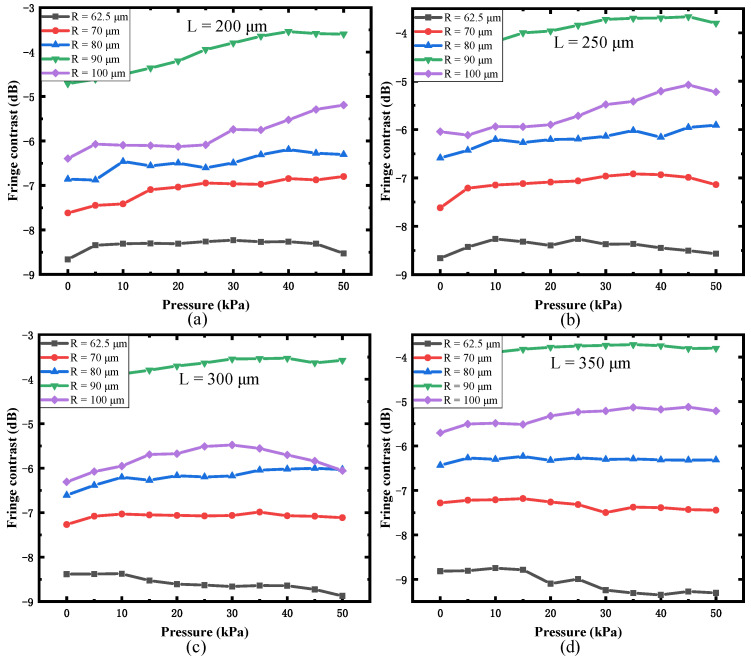
Relationship between the fringe contrast and applied pressure under different initial cavity lengths: (**a**) L = 200 μm, (**b**) L = 250 μm, (**c**) L = 300 μm, and (**d**) L = 350 μm.

The relationship between the fringe contrast and applied pressure was simulated with different initial cavity lengths L and curvature radii R, which is shown in Figure 9.

When the initial cavity L is fixed, with an increase in R, the fringe contrast increases and then decreases. The turning point is 90 μm. The fringe contrast is greatly improved with the increase in the curvature radius of the spherical end face R. When the curvature radius R is 90 μm, the signal contrast is the largest, which is about 4 dB higher than curvature radius R is 62.5 μm. With an increase in initial cavity length L, the fringe contrast only has a slight change. When the curvature radius R is 90 μm, the fringe contrast is slightly increased. In summary, the spherical end face can improve the fringe contrast. The best radius of the spherical end face is 90 μm. 

According to simulation results, the structural parameters of the sensor are selected as follows: the inner diameter of quartz capillary is 180 μm, the radius of silicone rubber diaphragm is 90 μm, and the thickness is 250 μm.

## 4. Sensor Fabrication

**Figure 10:**
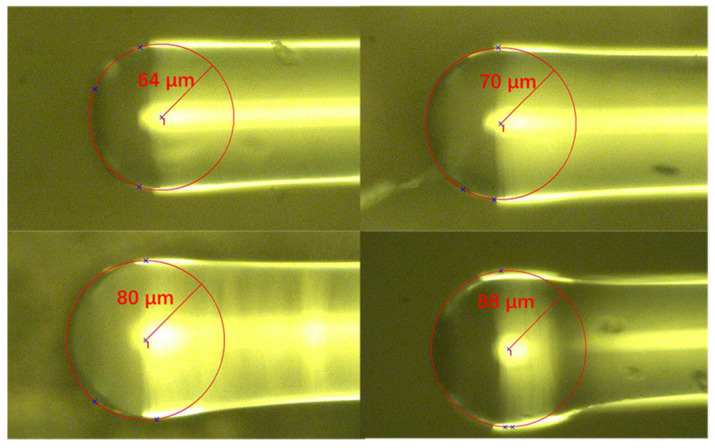
Spherical end faces with different radii.

To fabricate the proposed pressure sensor, we used a fusion splicer (FITEL, type: S179) to melt a fiber end face to form a spherical ball. Fusion time and discharge intensity were adjusted, and after multiple discharges, we obtained spherical balls with different radii, which are shown in Figure 10.

**Figure 11:**
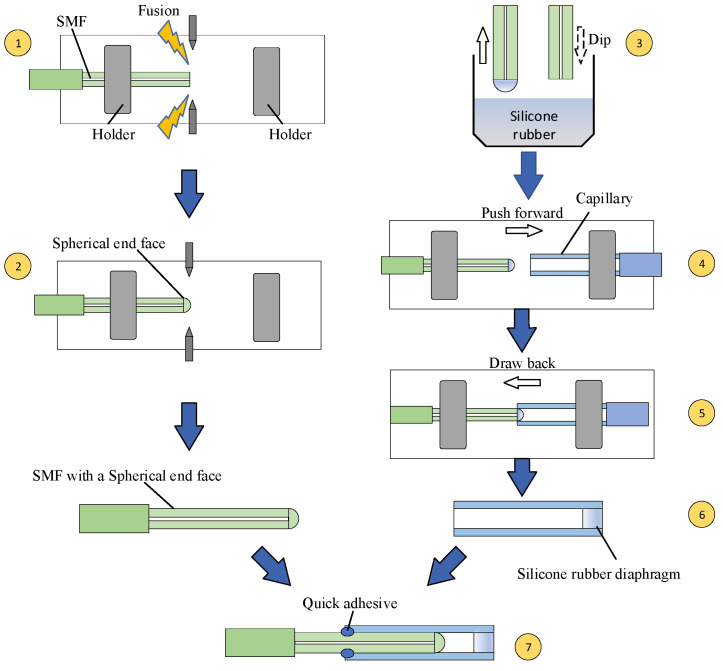
The fabrication process of proposed sensor.

**Figure 12:**
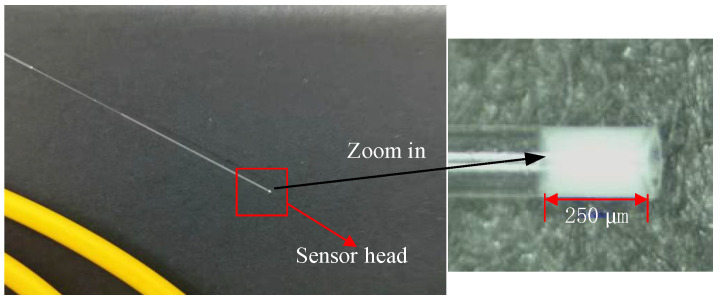
A pressure sensor sample.

Then, we began to fabricate a silicone rubber diaphragm; the fabrication process is given in Figure 11. First, silicone rubber components A and B were prepared, according to a mass ratio of 8:1; a small amount of liquid silicone rubber material was dipped onto the optical fiber, and the optical fiber was pushed forward by controlling the motor of the fusion splicer, and the liquid rubber was sucked into a quartz capillary by capillarity; then, the fiber was withdrawn back. The liquid rubber was allowed to cure in the quartz capillary for 24 h. A fiber with a spherical end fiber was then inserted into the capillary and sealed with quick adhesive at the end of the capillary. The sensor sample made according to this fabrication process is shown in Figure 12.

**Figure 13:**
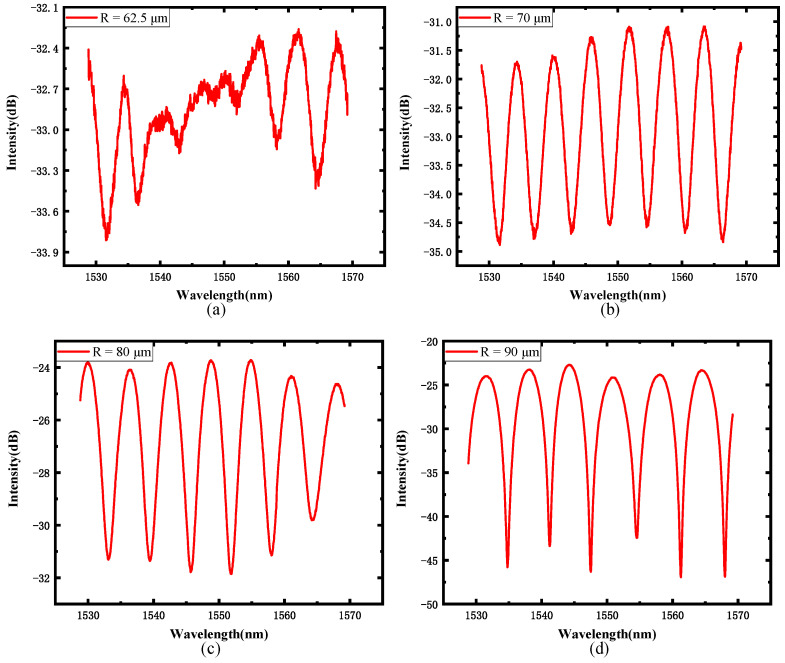
Sensors’ spectra: (**a**) radius is 62.5 μm, (**b**) radius is 70 μm, (**c**) radius is 80 μm, and (**d**) radius is 90 μm.

Four pressure sensors with different curvature radii of spherical end faces were fabricated, and their interference spectra are given in Figure 13. As the curvature radius increases, the fringe contrast indeed increases. The signal-to-noise ratio is greatly improved because the noise levels progressively decrease. When the curvature radius of the spherical end face is 90 μm, the fringe contrast is around 20 dB. Results are consistent with the simulation.

## 5. Experiments and Results

**Figure 14:**
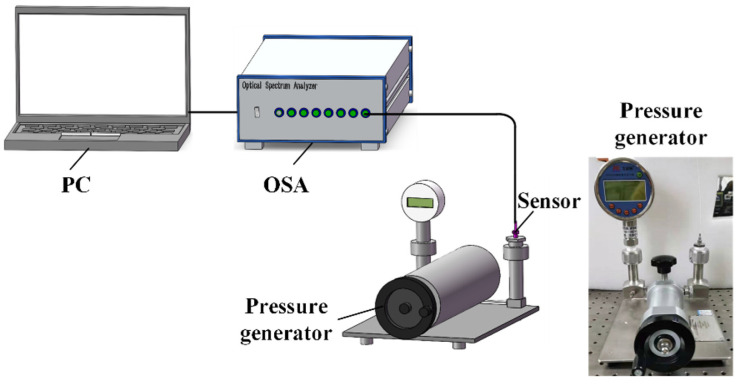
Experimental setup for pressure test.

To test the pressure response of our proposed sensor, an experiment system was set up, as shown in Figure 14. A proposed sensor sample was mounted to an air pressure generator (AILEIKE, ALKC400D); the pressure range is −100~100 kPa, and the accuracy is 10 Pa by a homemade connector converter. The fiber pigtail of the sensor was then connected to an optical spectrum analyzer, which had a build-in broadband light source in the range of 1520~1560 nm with a wavelength resolution of 10 pm.

**Figure 15:**
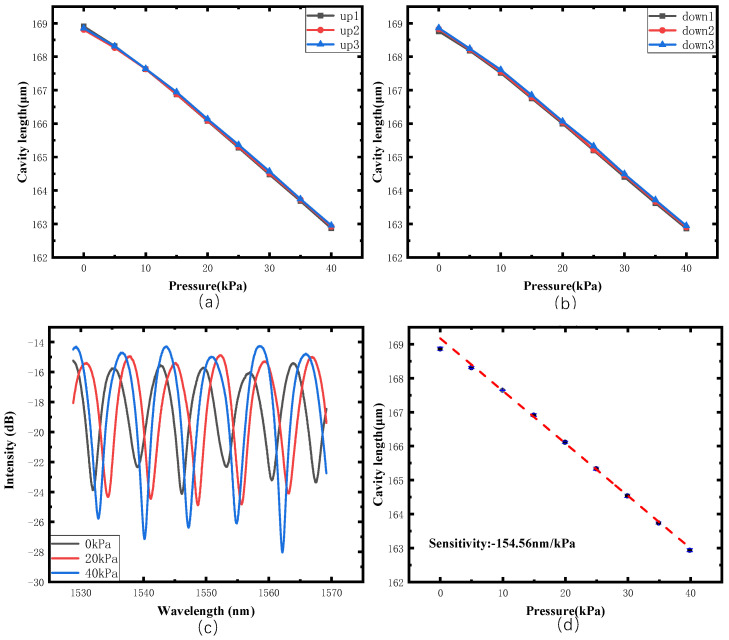
Response of FP cavity length to pressure. (**a**) relationship between the cavity length change and pressure in uploading process, (**b**) relationship between the cavity length change and pressure in downloading process, (**c**) spectrum changes during the pressure loading process, (**d**) a linear fitting of experimental results.

The experiments were conducted three times by increasing and decreasing the pressure in the range of 0~40 kPa with a step of 5 kPa. The pressure results are plotted in Figure 15. Figure 15c shows the spectrum changes during the pressure loading process. Figure 15a,b show the relationship between the cavity length change and pressure in uploading and downloading processes, respectively. To analyze the pressure response properties of the sensor, a linear fitting was taken according to the results given in Figure 15a,b. We can see that our proposed senor has good linearity in the pressure response with an R^2^ value of 0.998 and a sensitivity of −154.56 nm/kPa, which is around 100 times larger than reported results [20].

**Figure 16:**
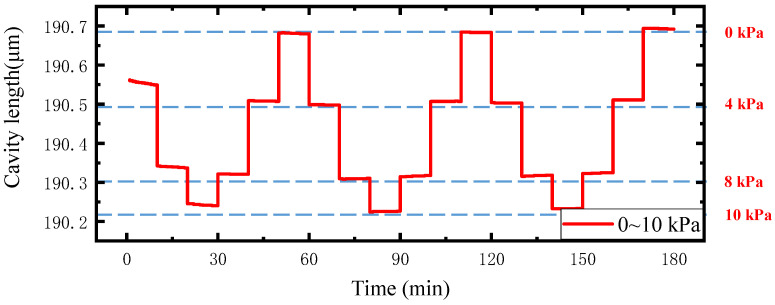
Stability test results.

**Figure 17:**
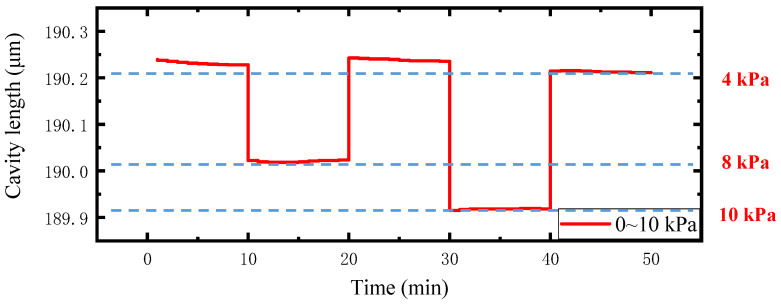
Test results after 7 days.

To investigate the long-term stability of the proposed sensor, an experiment was carried out in the pressure range of 0~10 kPa, during holding the pressure for 10 min under each spectrum data recording. The test results are shown in Figure 16. Then, we repeated the test again after 7 days, and the results are presented in Figure 17. The combined results of Figure 16 and Figure 17 reveal that the sensor has good stability and repeatability.

**Figure 18:**
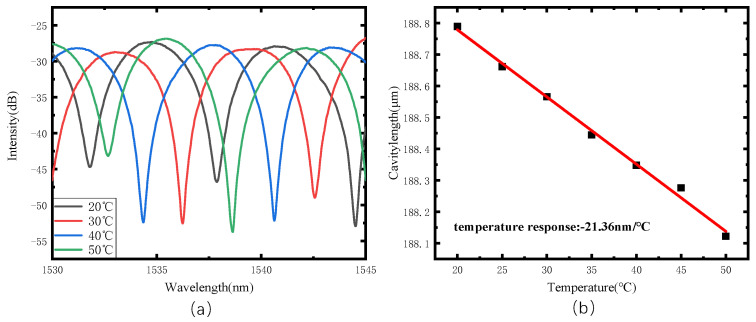
Temperature test results. (**a**) Temperature change response spectra and (**b**) temperature response.

The water bath method was used to investigate the response of the sensor to the change in temperature. During raising the temperature from 20 ℃ to 50 ℃ in a water bath, sensor spectra were recorded, as shown in Figure 18. When the temperature increases, although the silicone rubber expands, the cavity length decreases, and the temperature sensitivity is −21.36 nm/°C. Its corresponding pressure measurement error caused by temperature is around 0.143 kPa/°C, which is far lower than the results reported in previous studies [20].

## 6. Conclusions

In summary, a silicone rubber FP pressure sensor based on a spherical optical fiber end face was designed. The parameters of the proposed sensor were optimized by investigating the ratio of silicone rubber ingredients, the diameter, and thickness of the silicone rubber diaphragm, the radius of a spherical optical fiber, and initial cavity length. The spectra of fabricated samples show that our proposed sensors with a 90 μm radius spherical fiber end face have a good fringe contrast.

Gas pressure experimental results within a pressure range of 0~40 kPa show that the average sensitivity of the sensor is −154.56 nm/kPa, and repeatability error is less than 0.71%. Long-term pressure experimental results show that it has good repeatability and stability. Temperature experimental results show that its temperature cross-sensitivity is 0.143 kPa/°C. The good performance of the proposed FP sensor will expand its applications to biochemical applications, especially human body pressure monitoring.

## Figures and Tables

**Table 1:** Elastic coefficients of the silicone rubber.

Mass Ratio (M)	*C*_10_	*C*_01_
6:1	0.18419	−0.12967
8:1	0.28905	−0.14036
10:1	0.42135	−0.15127

## Data Availability

Not applicable.
